# Effects of Pneumococcal Conjugate Vaccine on Genotypic Penicillin Resistance and Serotype Changes, Japan, 2010–2017

**DOI:** 10.3201/eid2411.180326

**Published:** 2018-11

**Authors:** Kimiko Ubukata, Misako Takata, Miyuki Morozumi, Naoko Chiba, Takeaki Wajima, Shigeo Hanada, Michi Shouji, Megumi Sakuma, Satoshi Iwata, the Invasive Pneumococcal Diseases Surveillance Study Group

**Affiliations:** Keio University School of Medicine, Tokyo, Japan (K. Ubukata, M. Takata, M. Morozumi, N. Chiba, M. Sakuma, S. Iwata);; Tokyo University of Pharmacy and Life Sciences, Tokyo (T. Wajima);; Toranomon Hospital, Tokyo (S. Hanada);; National Cancer Center Hospital, Tokyo (M. Shouji, S. Iwata)

**Keywords:** invasive pneumococcal disease, serotype, antimicrobial resistance, *Streptococcus pneumoniae*, pneumococcal conjugate vaccine, multilocus sequence typing, bacteria, Japan

## Abstract

To clarify year-to-year changes in capsular serotypes, resistance genotypes, and multilocus sequence types of *Streptococcus pneumoniae*, we compared isolates collected from patients with invasive pneumococcal disease before and after introductions of 7- and 13-valent pneumococcal conjugate vaccines (PCV7 and PVC13, respectively). From April 2010 through March 2017, we collected 2,856 isolates from children and adults throughout Japan. Proportions of PCV13 serotypes among children decreased from 89.0% in fiscal year 2010 to 12.1% in fiscal year 2016 and among adults from 74.1% to 36.2%. Although nonvaccine serotypes increased after introduction of PCV13, genotypic penicillin resistance decreased from 54.3% in 2010 to 11.2% in 2016 among children and from 32.4% to 15.5% among adults. However, genotypic penicillin resistance emerged in 9 nonvaccine serotypes, but not 15A and 35B. Multilocus sequence typing suggested that resistant strains among nonvaccine serotypes may have evolved from clonal complexes 156 and 81. A more broadly effective vaccine is needed.

Among persons in all age groups, but particularly infants and elderly persons, *Streptococcus pneumoniae* remains a major cause of invasive pneumococcal disease (IPD) (e.g., pneumonia, meningitis, and sepsis), although generally effective antimicrobial agents are available (*1*). In the United States, 7-valent pneumococcal conjugate vaccine (PCV7) has been administered to children since 2000, resulting in both individual and herd immunity, with declines in pneumococcal infection among children and elderly persons (*2**–**6*). Unfortunately, introduction of PCV7 was followed by an increase in serotype 19A showing penicillin resistance and often multidrug resistance (*5**–**8*). In 2010, vaccination for children was upgraded to 13-valent pneumococcal conjugate vaccine (PCV13), which covers 6 additional serotypes: 1, 3, 5, 6A, 7F, and 19A (*9*). Introduction of PCV13 contributed to decreases in IPD (*10**,**11*), pneumonia (including community-acquired pneumonia without bacteremia) (*12**,**13*), and acute otitis media (*14**–**16*) caused by *S. pneumoniae* belonging to vaccine serotypes, especially 6A and 19A. As an indirect effect of wide administration of PCVs to children, pneumococcal infections in adults have also decreased, representing herd immunity (*11**,**12**,**17**–**23*).

Despite these benefits, in countries where PCV7 or PCV13 was introduced, proportions of disease preventable by PCVs gradually decreased because vaccine-serotype pneumococci were replaced by nonvaccine serotypes (NVTs). Increases in NVTs such as 6C, 15A/B/C, 23A, and 35B have been reported in the United States (*24**–**28*); 15A and 23B in Norway (*18*) and Germany (*29*); and 12F, 15A, 24F, and 35B in France (*30*).

In November 2010 in Japan, PCV7 vaccination use among children <5 years of age was introduced voluntarily by the Provisional Special Fund for the Urgent Promotion of Vaccination. In April 2013, PCV7 was officially incorporated into the vaccination program as public administration; in November of that year, PCV7 was replaced by PCV13. Promotion of PCV7 vaccination for children rapidly halved the number of IPD cases caused by vaccine-serotype pneumococci among children (*31*) and also produced a herd effect benefiting elderly persons (*32*). After PCV7 introduction, however, among persons of all ages, IPD caused by non-PCV7 serotypes such as 19A, 15A, 15B, 15C, 22F, and 24F showed relative increases in 2013. In November 2014, the Japanese Ministry of Health, Labour and Welfare began promoting vaccination of adults >65 years of age with 23-valent pneumococcal polysaccharide vaccine (PPSV23). In this study, we aimed to clarify year-to-year changes in capsular serotypes, genotypes of penicillin and macrolide resistance, and diversity of sequence types (STs) in all pneumococcal isolates collected throughout Japan during April 2010–March 2017.

## Methods

### Patients and Pneumococcal Strains

We included all specimens from patients of any age with IPD. Pneumococcal isolates from normally sterile clinical samples were collected from clinical laboratories at 341 hospitals participating in this IPD surveillance study. Each hospital had a microbiology laboratory as described previously (*31*), and participating hospitals were distributed nearly uniformly throughout Japan. These hospitals took part in the surveillance project after written permission was granted by the laboratory director or hospital director. This study was approved by the Keio University School of Medicine Ethics Committee (approval no. 20140432).

A total of 2,856 pneumococcal strains were collected from April 2010 through March 2017 Technical Appendix Figure 1. The first surveillance interval, April 2010–March 2011 (designated 2010), represented the pre-PCV7 period. The second surveillance interval, April 2011–March 2014 (designated 2011–2013, the PCV7 period), showed effects of PCV7 vaccination for children <5 years of age. The third surveillance interval, April 2014–March 2017 (designated 2014–2016, the PCV13 period), reflected PCV13 vaccination for children <5 years of age.

During the pre-PCV7 period, the rate of voluntary PCV7 vaccination among children in Japan was <10%. The PCV7 period corresponded to the Urgent Promotion of PCV7, a vaccination incentive program for children. The PCV7 vaccination rate throughout Japan was estimated at 50% –60% in 2011, 80%– 90% in 2012, and >95% in 2013. During the PCV13 period, corresponding to substitution of PCV13 for routine vaccination, coverage remained >95%. In elderly persons (>65 years of age), the rate of vaccination with PPSV23, starting in 2014, has remained at ≈54% as of 2017 (Vaccine Medical Affairs of Merck Sharp and Dohme K.K., Tokyo, Japan, pers. comm., 2017 Apr 1).

Pneumococcal isolates were sent promptly from each clinical laboratory to the Department of Infectious Diseases, Keio University School of Medicine (Tokyo, Japan), accompanied by a survey form completed by the attending physician. In compliance with ethics guidelines for epidemiology in Japan, patients were not identified.

### Serotype and Resistance Genotype

We determined serotypes by using the capsular quellung test with antiserum purchased from Statens Serum Institute (Copenhagen, Denmark). Alterations in 3 penicillin-binding protein genes that mediate β-lactam resistance in *S. pneumoniae* (*pbp1a*, *pbp2x*, and *pbp2b*) were identified by real-time PCR as described previously (*33*). The *mef*(A) and *erm*(B) genes, which mediate macrolide resistance, were also identified by real-time PCR (*33*). Quinolone resistance was analyzed by sequencing the quinolone resistance–determining region in the genes *gyrA*, *gyrB*, *parC*, and *parE* in strains showing MICs of levofloxacin exceeding 4 μg/mL.

Genotypes (g) based on gene analysis were represented as follows: penicillin-susceptible *S. pneumoniae* (gPSSP), possessing 3 normal *pbp* genes; penicillin-intermediate *S. pneumoniae* (gPISP), subclassified as gPISP (*pbp2x*), gPISP (*pbp2b*), gPISP (*pbp1a+pbp2x*), gPISP (*pbp1a+pbp2b*), or gPISP (*pbp2x+pbp2b*); and penicillin-resistant *S. pneumoniae* (gPRSP), which possessed 3 abnormal *pbp* genes (*31**,**33*). Serotype and resistance genotype results were promptly reported to laboratory staff at each referring hospital.

### Susceptibility Testing

For all isolates, we redetermined the MICs of 6 antimicrobial agents by using agar-dilution methods with reference strains R6 and ATCC49619 (*34*). The agents tested were penicillin, ampicillin, cefotaxime, meropenem, vancomycin, and levofloxacin.

### Multilocus Sequence Typing

We performed multilocus sequence typing (MLST) analysis for all 2,849 isolates that could be cultured. Primers used for MLST were based on sequences listed at https://pubmlst.org/spneumoniae/. Clusters of related STs were analyzed by using eBURST version 3 (http://eburst.mlst.net/).

### Statistical Analyses

For statistical analyses, we used Ekuseru-Toukei 2015 software (Social Survey Research Information, Tokyo, Japan) and R software 3.5.0 (R Foundation of Computational Statistics, Vienna, Austria). We used the χ^2^ and Fisher exact tests as appropriate. We considered p<0.05 to indicate statistical significance.

## Results

### Relationships between IPD Type and Patient Age

Relationships between IPD type and patient age are shown in Table 1. IPD types were classified into 4 categories: pneumonia with bacteremia (41.9%), including empyema and pleuritis; bacteremia with unknown focus (37.0%); meningitis (15.4%); and others (5.6%), including endocarditis, necrotizing fasciitis, cellulitis, arthritis, and spondylitis. Pneumonia with bacteremia was most common among adults, especially those >75 years of age; however, among children <5 years of age, bacteremia with unknown focus was most common (p<0.001 for each). Meningitis and other IPDs were represented in higher proportions among persons 6–64 years of age (p<0.001) than among those in other age groups (p = 0.002).

**Table 1:** Invasive pneumococcal disease in all patients, by age group, Japan, April 2010–March 2017

Disease	Total, no. (%), n = 2**,**856	Age, y, no. (%)								p value
		<2, n = 731	3–5, n = 181	6–17, n = 94	18–49, n = 201	50–64, n = 387	65–74, n = 530	75–84, n = 457	>85, n = 275	
Pneumonia with bacteremia*	1,198 (41.9)	130 (17.8)	35 (19.3)	22 (23.4)	83 (41.3)	167 (43.2)	261 (49.2)	300 (65.6)	200 (72.7)	<0.001
Bacteremia with focus unknown	1,058 (37.0)	455 (62.2)	116 (64.1)	33 (35.1)	46 (22.9)	104 (26.9)	158 (29.8)	92 (20.1)	54 (19.6)	<0.001
Meningitis	440 (15.4)	109 (14.9)	22 (12.2)	34 (36.2)	56 (27.9)	80 (20.7)	79 (14.9)	43 (9.4)	17 (6.2)	<0.001
Other†	160 (5.6)	37 (5.1)	8 (4.4)	5 (5.3)	16 (8.0)	36 (9.3)	32 (6.0)	22 (4.8)	4(1.5)	0.002

*Includes empyema (n = 32) and pleuritis (n = 25). †Includes endocarditis (n = 6), necrotizing fasciitis (n = 1), arthritis (n = 34), cellulitis (n = 20), and spondylitis (n = 7).

### Changes in Serotypes

**Figure 1:**
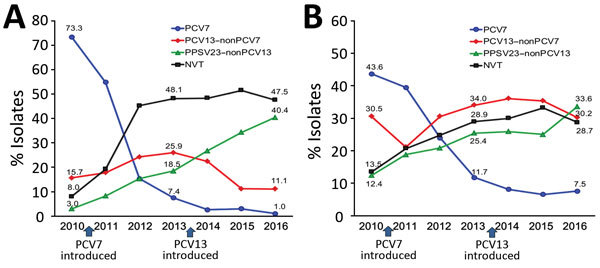
Yearly changes in pneumococcal serotypes of isolates from A) 1,006 children and B) 1,850 adults with invasive pneumococcal disease in Japan, April 2010–March 2017. Specific percentages are indicated at points along data lines. Fiscal years extend from April 1 through March 31 of the following year. PCV13–nonPCV7 covers 6 serotypes (1, 3, 5, 6A, 7F, and 19A). PPSV23–nonPCV13 covers 11 serotypes (2, 8, 9N, 10A, 11A, 12F, 15B, 17F, 20, 22F, and 33F), but 2, 9N, and 17F were not isolated in this study. NVTs represent other serotypes not included in PPSV23 and 6A. NVT, nonvaccine serotype; PCV7, 7-valent pneumococcal conjugate vaccine; PCV13, 13-valent pneumococcal conjugate vaccine; PPSV23, 23-valent pneumococcal polysaccharide vaccine.

Figure 1 shows yearly changes in pneumococcal capsular serotypes among children and adults. Pneumococcal capsular serotypes were classified into 4 groups: PCV7 serotypes (4, 6B, 9V, 14, 18C, 19F, and 23F) (PCV7); PCV13 serotypes not included in PCV7 (1, 3, 5, 6A, 7F, and 19A) (PCV13–nonPCV7); PPSV23 serotypes not included in PCV13 (2, 8, 9N, 10A, 11A, 12F, 15B, 17F, 20, 22F, and 33F) (PPSV23–nonPCV13); and NVTs not including serotypes in PPSV23 and not including 6A. Among children, the proportion of PCV7 serotypes that accounted for 73.3% of serotype strains isolated from IPD patients during the pre-PCV7 period decreased rapidly to 7.4% in 2013 after PCV7 introduction (Figure 1). In contrast, in 2013, PCV13–nonPCV7 serotypes increased from 15.7% to 25.9%, PPSV23–nonPCV13 serotypes increased from 3.0% to 18.5%, and NVT serotypes increased from 8.0% to 48.1%. During 2014, after PCV7 was replaced with PCV13, the proportion of PCV13–nonPCV7 serotypes decreased by approximately half to 11.1% in 2016, while PPSV23–nonPCV13 serotypes increased to 40.4%, in contrast to the PCV7 period. 

Among adults, the proportions of PCV7 serotypes, which accounted for 43.6% of isolates during the pre-PCV7 period, decreased to 11.7% in 2013, when children were vaccinated with PCV7. However, proportions of the PPSV23–nonPCV13 doubled from 12.4% to 25.4% and NVTs doubled from 13.5% to 28.9%. PCV13–nonPCV7 serotypes decreased slightly after replacement by PCV13 in 2014, but PPSV23–nonPCV13 serotypes continued to increase.

### Serotype Changes during the Pre-PCV7, PCV7, and PCV13 Periods

Changes in serotypes of pneumococcal isolates collected between the pre-PCV7, PCV7, and PCV13 periods are shown in Table 2 for children and in Table 3 for adults. Among children, proportions of PCV7 serotypes decreased rapidly from 73.3% to 30.3% during the PCV7 period and decreased further to 2.3% during the PCV13 period (p<0.001). Among PCV13–nonPCV7 serotypes, serotype 19A apparently increased during the PCV7 period, but later it decreased significantly during the PCV13 period. PCV13–nonPCV7 serotypes decreased from 21.8% during the PCV7 period to 14.9% during the PCV13 period (p = 0.031). Although serotypes 1 and 7F showed relative increases during the PCV13 period, most were isolated from patients >3 years of age who had received PCV7 or a single dose of PCV13. To the contrary, proportions of PPSV23–nonPCV13 serotypes and NVTs increased significantly between the pre-PCV7 period and the PCV7 period, continuing to increase up to the PCV13 period (p<0.001 for each). In particular, 9 serotypes (10A, 12F, 15A, 15B, 15C, 22F, 24F, 33F, and 35B) increased significantly after introduction of PCV7 and PCV13.

**Table 2:** Distribution of pneumococcal serotypes among children before PCV7 and after introduction of PCV7 and PCV13, Japan, April 2010–March 2017*

Serotype	No. (%)			p value†
	Pre-PCV7 period**,** 2010, n = 300	PCV7 period 2011–2013, n = 357	PCV13 period**,** 2014–2016, n = 349	
PCV7				
4	7 (2.3)	6 (1.7)	0	0.007
6B	83 (27.7)	49 (13.7)	2 (0.6)	<0.001
9V	9 (3.0)	2 (0.6)	1 (0.3)	0.005
14	34 (11.3)	9 (2.5)	0	<0.001
18C	4 (1.3)	4 (1.1)	1 (0.3)	0.336
19F	40 (13.3)	13 (3.6)	2 (0.6)	<0.001
23F	43 (14.3)	25 (7.0)	2 (0.6)	<0.001
Subtotal	220 (73.3)	108 (30.3)	8 (2.3)	<0.001
PCV13–nonPCV7‡				
1	0	5 (1.4)	13 (3.7)	**<0.001**
3	4 (1.3)	7 (2.0)	4 (1.1)	0.724
5	0	0	0	NA
6A	15 (5.0)	6 (1.7)	2 (0.6)	0.001
7F	1 (0.3)	2 (0.6)	6 (1.7)	0.153
19A	27 (9.0)	58 (16.2)	27 (7.7)	0.001
Subtotal	47 (15.7)	78 (21.8)	52 (14.9)	0.031
PPSV23–nonPCV13§				
8	0	0	0	NA
10A	2 (0.7)	5 (1.4)	18 (5.2)	**<0.001**
11A	2 (0.7)	1 (0.3)	4 (1.1)	0.356
12F	1 (0.3)	1 (0.3)	33 (9.5)	**<0.001**
15B	0	15 (4.2)	26 (7.4)	**<0.001**
20	0	1 (0.3)	0	NA
22F	3 (1.0)	16 (4.5)	19 (5.4)	**0.003**
33F	1 (0.3)	7 (2.0)	17 (4.9)	**0.001**
Subtotal	9 (3.0)	46 (12.9)	117 (33.5)	**<0.001**
NVT				
6C	9 (3.0)	20 (5.6)	11 (3.2)	0.159
15A	2 (0.7)	25 (7.0)	36 (10.3)	**<0.001**
15C	1 (0.3)	17 (4.8)	16 (4.6)	**<0.001**
23A	4 (1.3)	13 (3.6)	10 (2.9)	0.176
24F	1 (0.3)	20 (5.6)	52 (14.9)	**<0.001**
24B	2 (0.7)	4 (1.1)	12 (3.4)	0.197
34	1 (0.3)	6 (1.7)	7 (2.0)	0.126
35B	1 (0.3)	10 (2.8)	11 (3.2)	**0.016**
38	1 (0.3)	8 (2.2)	7 (2.0)	0.072
Other¶	2 (0.7)	2 (0.6)	9 (2.6)	**0.041**
Subtotal	24 (8.0)	125 (35.0)	171 (49.0)#	**<0.001**

*Years run from April 1 through March 31 of the following year. NA, not applicable; NVT, nonvaccine serotype; PCV7, 7-valent pneumococcal conjugate vaccine; PCV13, 13-valent pneumococcal conjugate vaccine; PPSV23, 23-valent pneumococcal polysaccharide vaccine. †p values compare the 3 surveillance periods; boldface indicates significant increase.. ‡Serotypes added to PCV7.  §Serotypes contained in PPSV23 but not PCV13. ¶Includes 7C (n = 2), 16F (n = 2), 21 (n = 2), 23B (n = 3), 28A (n = 1), 37 (n = 1), and 31 (n = 2). #One strain identified as nontypeable was excluded from the table.

**Table 3:** Distribution of pneumococcal serotypes in adults before PCV7 and after introduction of PCV7 and PCV13 administration to children, Japan, April 2010–March 2017*

Serotype	No. (%)			p value†
	Pre-PCV7 period, 2010, n = 275	PCV7 period, 2011–2013, n = 695	PCV13 period, 2014–2016, n = 880	
PCV7				
4	14 (5.1)	27 (3.9)	4 (0.5)	<0.001
6B	42 (15.3)	39 (5.6)	22 (2.5)	<0.001
9V	7 (2.5)	7 (1.0)	6 (0.7)	0.042
14	21 (7.6)	41 (5.9)	6 (0.7)	<0.001
18C	1 (0.4)	3 (0.4)	2 (0.2)	0.642
19F	14 (5.1)	23 (3.3)	15 (1.7)	0.007
23F	21 (7.6)	28 (4.0)	9 (1.0)	<0.001
Subtotal	120 (43.6)	168 (24.2)	64 (7.3)	<0.001
PCV13–nonPCV7‡				
1	1 (0.4)	4 (0.6)	13 (1.5)	0.145
3	45 (16.4)	110 (15.8)	145 (16.5)	0.939
5	0	1 (0.1)	0	NA
6A	11 (4.0)	16 (2.3)	9 (1.0)	0.006
7F	9 (3.3)	9 (1.3)	33 (3.8)	**0.006**
19A	18 (6.5)	61 (8.8)	99 (11.3)	**0.045**
Subtotal	84 (30.5)	201 (28.9)	299 (34.0)	0.093
PPSV23–nonPCV13§				
8	0	2 (0.3)	0	NA
10A	10 (3.6)	34 (4.9)	54 (6.1)	0.244
11A	3 (1.1)	23 (3.3)	34 (3.9)	0.058
12F	5 (1.8)	5 (0.7)	63 (7.2)	**<0.001**
15B	3 (1.1)	14 (2.0)	10 (1.1)	0.356
20	1 (0.4)	7 (1.0)	14 (1.6)	0.261
22F	10 (3.6)	63 (9.1)	59 (6.7)	**0.008**
33F	2 (0.7)	4 (0.6)	11 (1.3)	0.352
Subtotal	34 (12.4)	152 (21.9)	245 (27.8)	**<0.001**
NVT				
6C	13 (4.7)	49 (7.1)	52 (5.9)	0.400
15A	6 (2.2)	28 (4.0)	47 (5.3)	0.068
15C	0	12 (1.7)	7 (0.8)	**0.034**
23A	2 (0.7)	33 (4.7)	50 (5.7)	**<0.001**
24F	0	11 (1.6)	16 (1.8)	**0.049**
34	1 (0.4)	5 (0.7)	12 (1.4)	0.301
35B	7 (2.5)	22 (3.2)	55 (6.3)	**0.004**
38	3 (1.1)	7 (1.0)	11 (1.3)	0.955
Other¶	5 (1.8)	6 (0.9)	22 (2.5)	**0.042**
Subtotal	37 (13.5)	173 (24.9)#	272 (30.9)	**<0.001**

*Years run April 1– March 31 of the following year. NA, not applicable; NVT, nonvaccine serotype; PCV7, 7-valent pneumococcal conjugate vaccine; PCV13, 13-valent pneumococcal conjugate vaccine; PPSV23, 23-valent pneumococcal polysaccharide vaccine. †p values compare the 3 surveillance periods; boldface indicates significant increase. ‡Serotypes added to PCV7.  §Serotypes contained in PPSV23 but not PCV13. ¶Includes 6D (n = 2), 7C (n = 8), 13 (n = 1), 16F (n = 6), 18B (n = 1), 23B (n = 5), 31 (n = 3), and 37 (n = 7). #One strain identified as nontypeable was excluded from the table.

Among adults, proportions of PCV7 serotypes decreased sharply, from 43.6% during the pre-PCV7 period to 24.2% during the PCV7 period and 7.3% during the PCV13 period, particularly for serotypes 4, 6B, 9V, 14, 19F, and 23F. PCV13–nonPCV7 serotypes increased in serotypes 7F and 19A, whereas 6A showed a significant decrease because of cross-immunity with 6B (Table 3). PPSV23–nonPCV13 serotypes and NVTs increased respectively from 12.4% and 13.5% during the pre-PCV7 period to 21.9% and 24.9% during the PCV7 period and further to 27.8% and 30.9% during the PCV13 period (p<0.001 for each). In particular, significant increases were noted for serotypes 12F, 15C, 22F, 23A, 24F, and 35B. Tendencies to increase did not attain significance for serotypes 11A and 15A.

### Changes in Penicillin and Other Resistance Genotypes

**Figure 2:**
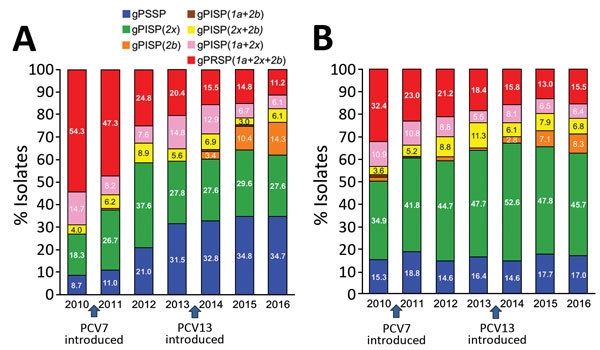
Yearly changes in genotypic penicillin resistance in isolates from A) 1,006 children and B) 1,850 adults with invasive pneumococcal disease in Japan, April 2010–March 2017. Fiscal years extend from April 1 through March 31 of the following year. Genotypes based on abnormal *pbp1a*, *pbp2x*, and *pbp2b* genes were identified by real-time PCR and are represented as gPRSP (*1a+2x+2b*), gPISP (*1a+2x*)*,* gPISP (*1a+2b),* gPISP (*2x+2b),* gPISP (*2x*)*,* gPISP (*2b*)*,* and gPSSP. g, genotype; PCV7, 7-valent pneumococcal conjugate vaccine; PCV13, 13-valent pneumococcal conjugate vaccine; PISP, penicillin-intermediate *Streptococcus pneumoniae;* PRSP, penicillin-resistant *S. pneumoniae*; PSSP, penicillin-susceptible *S. pneumoniae*.

Figure 2 shows yearly changes of penicillin resistance genotypes among children and adults. Changes are shown from the pre-PCV7 period to the PCV7 period and further to the PCV13 period.

Among children, the proportion of gPRSP declined sharply from 54.3% in 2010 during the pre-PCV7 period to 20.4% in 2013 during the PCV7 period; gPSSP and gPISP (*pbp2x*) increased (Figure 2). In 2016 during the PCV13 period, proportions of gPRSP and gPISP (*pbp1a+2x*) further declined to 11.2% and 6.1%, respectively. However, gPISP (*pbp2b*) rapidly increased.

Among isolates from adults during the pre-PCV7 period, gPISP (*pbp2x*) was most common (34.9%), followed by gPRSP (32.4%) (Figure 2). gPSSP accounted for only 15.3%. Similar to the trend for children during the PCV7 and PCV13 periods, gPRSP among adults continually decreased to 15.5% in 2016. However, also in 2016, gPISP (*pbp2b*) among adults increased to 8.3%, similar to the trend among children.

During the surveillance periods, macrolide-resistant isolates possessing *mef*(A) or *erm*(B) genes remained consistently high. Among children, proportions were 93.8% in 2010 and 91.8% in 2016; among adults, proportions were 87.2% in 2010 and 89.8% in 2016. Prevalence of resistance genes was 59.8% for the *erm*(B) gene mediating high macrolide resistance, 19.6% for the *mef*(A) gene mediating intermediate resistance, and 11.6% for both *erm*(B) and *mef*(A) genes (data not shown).

Isolates with mutations in both *gyrA* and *parC* genes, which are involved in resistance to quinolones, especially levofloxacin, accounted for <1% of all isolates. These isolates showed no tendency to increase.

### Relationships between Serotypes and Resistance Genotypes

Changes of serotypes and the penicillin resistance genotypes during the 3 periods (pre-PCV7, PCV7, and PCV13) are shown in Technical Appendix Figures 2 (for children) and 3 (for adults). Decreases in gPRSP (*pbp1a+2x+2b*) and gPISP (*pbp1a+2x*) were closely related to reduction of serotypes 6B, 14, 19F, 23F, and 6A in children and adults during the PCV7 period, and this link became stronger during the PCV13 period. Serotype 19A, including several gPRSP, decreased by half among children during the PCV13 period, but this change has not yet become evident among adults.

The proportions of PPSV23–nonPCV13 and NVT serotypes generally increased among children and adults during the PCV13 period. gPRSPs were newly identified in serotypes 15B (n = 1), 15C (n = 1), and 16F (n = 2) in isolates from children and in serotypes 6C (n = 2), 6D (n = 2), 13 (n = 1), 15B (n = 1), 15C (n = 1), 16F (n = 2), 23A (n = 1), 23B (n = 1), and 34 (n = 1) in isolates from adults.

Relationships between genotypic macrolide and penicillin resistances and serotypes are shown in Technical Appendix Table 1. Strains possessing *mef*(A), *erm*(B), or both were identified in most of the serotypes, with the exception of serotypes 8, 18B, 28A, and 31. No relationship was observed between macrolide resistance and penicillin resistance.

### Antimicrobial Susceptibility by Genotype

Susceptibilities (50% MIC, 90% MIC, and MIC range) of 6 parenteral agents (penicillin, ampicillin, cefotaxime, meropenem, vancomycin, and levofloxacin) for *S. pneumoniae* strains obtained from April 2014 through March 2017, corresponding to the PCV13 period (n = 1,229), are shown in Technical Appendix Table 2. Relationships between 6 genotypes for penicillin resistance and MICs of penicillin, ampicillin, cefotaxime, and meropenem for the strains are shown in Technical Appendix Figure 4.

Because prevalence of gPRSP was reduced by the PCV vaccinations, the distribution of susceptibilities was shifted in favor of greater susceptibility, especially after introduction of PCV7. For penicillin and ampicillin, 90% MICs were 2 μg/mL; for cefotaxime, 1 μg/mL; and for meropenem, 0.5 μg/mL. gPRSP isolates showing high resistance for penicillin (>8 μg/mL) were not found.

### STs by Serotypes and Resistance Genotypes

**Figure 3:**
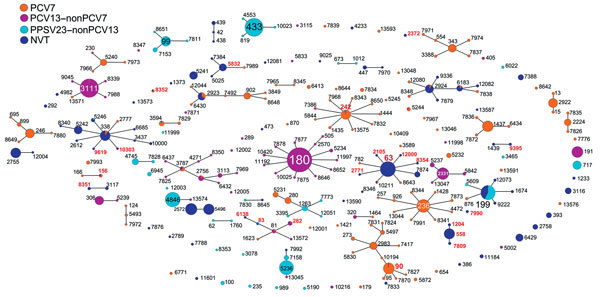
An eBURST (http://eburst.mlst.net/) diagram displaying pneumococcal sequence types (STs) causing invasive pneumococcal disease across patients of all age groups in Japan. All 2,849 strains are distinguished by colors to indicate PCV7, PCV13–nonPCV7, PPSV23–nonPCV13, and NVT. Size of each circle reflects the number of strains. ST numbers shown in red represent genotypes for penicillin-resistant *Streptococcus pneumoniae* confirmed among PPSV23–nonPCV13 and NVT as follows: 15B (n = 2), ST242 and ST83; 6C (n = 2), ST8352 and ST5832; 6D (n = 2), ST90 and ST282; 13 (n = 1), ST10303; 15A (n = 77), ST63 (n = 73), ST2105, ST2771, ST8354, and ST12000; 15C (n = 2), ST83 and ST6138; 16F (n = 4), ST8351; 23A (n = 1), ST9619; 23B (n = 1), ST2372; 34 (n = 1), ST9395; 35B (n = 55), ST558 (n = 49), ST1204, ST7809, ST7990, and ST156. NVT, nonvaccine serotype; PCV7, 7-valent pneumococcal conjugate vaccine; PCV13, 13-valent pneumococcal conjugate vaccine; PPSV23, 23-valent pneumococcal polysaccharide vaccine.

STs by eBURST analyses for 2,849 pneumococcal strains are shown in Figure 3. These data are distinguished by ST and vaccine serotype (PCV7, PCV13–nonPCV7, PPSV23–nonPCV13, and NVT). Details of relationships among clonal complexes (CCs), STs, serotypes, and resistance genotypes are listed in Technical Appendix Table 3.

By MLST analysis, 273 different STs were identified. STs of gPRSP in 11 serotypes included in PPSV23–nonPCV13 and NVTs were noteworthy: 15B (n = 2), ST242 (belonging to CC242) and ST83 (derived from CC81); 6C (n = 2), ST8352 (CC156) and ST5832 (CC5832); 6D (n = 2), ST90 (CC156) and ST282 (CC81); 13 (n = 1), ST10303 (CC156); 15A (n = 77), ST63, ST2105, ST2771, ST8354, and ST12000 (all CC63); 15C (n = 2), ST83 and ST6138 (CC81); 16F (n = 4), ST8351 (CC3117); 23A (n = 1), ST9619 (CC156); 23B (n = 1), ST2372 (CC156); 34 (n = 1), ST9395 (CC15); 35B (n = 55), ST558, ST1204, and ST7809 (all CC558); and the remaining ST156 (CC156) and ST7990 (singleton).

**Figure 4:**
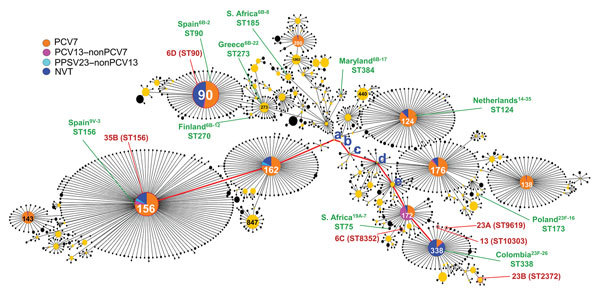
Details of *Streptococcus pneumoniae* clonal complex (CC) 156 (n = 4,736), including 1,308 sequence types obtained from the multilocus sequence typing website (https://pubmlst.org/spneumoniae/). Data include those from this study (n = 359). STs of 6 genotypes for penicillin-resistant *S. pneumoniae* identified in NVT serotypes belonged to CC156. STs of serotypes 6C, 13, 23A, and 23B were derived from ST338 and ST172 (shown in red). Serotypes 6D and 35B belonged to ST90 and ST156, respectively. The Pneumococcal Molecular Epidemiology Network clone identified in CC156 is also shown (in green). The red line indicates evolution from ST156 to ST338: a, ST8055; b, ST8618; c, ST4542; d, ST171; e, ST361. NVT, nonvaccine serotype; PCV7, 7-valent pneumococcal conjugate vaccine; PCV13, 13-valent pneumococcal conjugate vaccine; PPSV23, 23-valent pneumococcal polysaccharide vaccine; ST, sequence type.

A total of 6 STs identified as gPRSP belonged to the large CC156 (Figure 4). STs of 3 serotypes (13, 23A, and 23B) were derived from ST338, which includes the Colombia^23F-26^ clone from the Pneumococcal Molecular Epidemiology Network (PMEN). Serotype 6C was derived from ST172, a neighbor of ST338. Of strains with serotypes 6D and 35B, each strain was distant from other gPRSPs. STs of serotypes 15B, 15C, and 6D among gPRSP belonged to CC81 (Technical Appendix Figure 5).

In addition, STs of certain serotypes increasing in PPSV23–nonPCV13 and NVTs were noted. Serotype 12F was ST4846 (CC1527), 22F was ST433 (CC433), 23A included ST338 and ST5242 (CC156), and 24B/24F included ST2572 and ST5496 (CC2572).

## Discussion

Wide use of PCVs among children in many countries has contributed to a dramatic reduction in incidence of IPD (*6**,**10**,**11**,**35**–**38*), pneumonia (*12**,**13**,**39*), and acute otitis media (*14**,**15*) caused by *S. pneumoniae*, while providing indirect herd immunity benefits for adults (*11**,**23**,**40**,**41*). Replacing PCV7 with PCV13 decidedly decreased serotype 19A isolates among causative pathogens, but in several countries, NVTs such as 15A and 35B increased. Gradual increases of NVTs, unfortunately, have blunted the effectiveness of conjugate vaccines (*42*).

In Japan, introduction of PCV7 in children <5 years of age began as an official government program in November 2010, continuing until it was replaced with PCV13 in November 2013. PPSV23 vaccination for adults >65 years of age was implemented in October 2014. We organized nationwide surveillance beginning in April 2010, with collection of pneumococcal strains from IPD patients in all age groups throughout Japan. In this article, we describe details of changes of serotypes, penicillin resistance genotypes, and MLST analyses that have followed implementation of PCV7 and PCV13 vaccination. As in other countries where PCV13 has been introduced, proportions of PCV13 serotypes among isolates from children and adults decreased significantly during the PCV13 period. In Japan, where population density is high, the decrease suggests early effectiveness of herd immunity not only among children but also among adults. However, serotypes 7F and 19A, included in PCV13, seem to be increasing among adults; for these serotypes, no indirect effect for adults is evident. These findings indicate a need for PCV13 vaccination of elderly and relatively immunocompromised persons, especially in Japan where the populations average age is increasing rapidly. Of further concern is a test-negative design study conducted before introduction of PPSV23 to assess effectiveness of PPSV23 among elderly persons with community-acquired pneumonia in Japan. Effectiveness against community-acquired pneumonia caused by PPSV23 serotypes seemed low to moderate, depending on age group (*43*).

Proportions of many non-PCV13 serotypes during the PCV13 period have increased beyond proportions during the pre-PCV7 period. Nine serotypes (10A, 12F, 15A, 15B, 15C, 22F, 24F, 33F, and 35B) have increased significantly among children, and 5 serotypes (12F, 15C, 22F, 23A, and 35B) have increased significantly among adults, showing considerable overlap between age groups. Among these serotypes, 15A and 35B have increased rapidly since PCV13 introduction in Japan, as has occurred in other countries (*18**,**25**,**28**–**30*). The reason for increases in such serotypes is unclear; further epidemiologic surveillance may shed light on the matter.

Of note, gPRSP decreased sharply along with serotype replacements among children and adults. Highly penicillin-resistant strains with MICs >8 μg/mL, which sometimes were noted in serotypes 19F and 23F during the pre-PCV7 period (*33*), did not increase with introduction of PCVs. Susceptibilities of most gPISP (*pbp2b*) in serotype 12F and of gPISP(*pbp2x+2b*) in serotypes 23A and 6C for penicillin and ampicillin ranged from 0.125 to 0.5 μg/mL. Should mutation(s) occur in the regions encoding the conserved amino acids (STMK, SSN, and KTG) in the *pbp1a* gene, antimicrobial selection pressure could easily favor development from gPISP to gPRSP.

One concern is the evolution of gPRSP among isolates from 11 NVTs according to MLST analysis. Most (all but 2) serotype 35B isolates were found to belong to the same ST558 (CC558) that was reported from the United States in 1999 (*44*). Serotype 15A was identified as ST63, which belongs to CC63, as does the PMEN clone Sweden^15A-25^. Each isolate of serotype 6D (ST282, CC81) and serotype 15B (ST83, CC81) was the same as those previously registered from South Korea (*45*) and Taiwan. These findings suggest that newly emerged resistant strains can spread rapidly between countries.

Among gPRSP identified in NVTs, STs of serotypes 6C, 13, 23A, 23B, and of both serotypes 6D and 35B, were noted to belong to CC156, which includes large numbers of isolates in ST156, ST90, ST162, ST124, ST176, and ST138; the PMEN clones Spain^9V-3^, Netherlands^14-35^, Spain^6B-2^, Greece^6B-22^, and S. Africa^6B-8^ are representative among these. STs of serotypes 6C, 13, 23A, and 23B were derived from ST172 and ST338, which diverged from ST171 (Figure 4, letter d) and evolved further. Serotypes of many isolates registered as ST172 or ST338 were either NVTs or one of the serotypes of PCV13–nonPCV7. These findings suggested that wide use of PCVs led to a decrease in STs belonging to PCV7 and PCV13 serotypes, but some STs detected among NVTs escaped from the vaccine pressure and are increasing, such as ST338. However, whether the new gPRSPs emerged in Japan or originated in another country is unknown.

Capsular switching in *S. pneumoniae* can occur as a result of homologous recombination at a site outside the *cps* locus. Of note, *pbp1a* genes are located upstream and *pbp2x* genes are located downstream of the *cps* locus (*46**–**48*). Recombination including these 2 *pbp* genes, driven by antimicrobial pressure, can result in concomitant exchange of the *cps* locus. Such new ST strains arising from capsular switching can exhibit penicillin resistance and increase under antimicrobial selection pressure. The diversity of serotypes, resistant genotypes, and STs we describe reflects adaptability of *S. pneumoniae* to the human environment.

In conclusion, to assess whether gPRSP in NVTs will increase in the near future, sustained surveillance for IPD is needed. Control of pneumococcal infections, particularly in elderly and immunocompromised persons, requires development of further multivalent conjugate vaccines, new vaccines targeting a different microbial component, or both. Global consensus for appropriate use of antimicrobial drugs is also valuable for limiting spread of new resistant strains within and beyond national borders.

Technical Appendix Additional results from study of effects of pneumococcal conjugate vaccine on genotypic penicillin resistance and serotype changes, Japan, 2010–2017.
