# Supplementary material for: Effects of Pneumococcal Conjugate Vaccine on Genotypic Penicillin Resistance and Serotype Changes, Japan, 2010–2017
Source: Emerg Infect Dis. 2018 Nov;24(11):2010–20. doi: 10.3201/eid2411.180326 (PMC6200004; doi:10.3201/eid2411.180326)
Supplement: Technical Appendix — Additional results from study of effects of pneumococcal conjugate vaccine on genotypic penicillin resistance and serotype changes, Japan, 2010–2017. [file 18-0326-Techapp-s1.pdf]

Article DOI: https://doi.org/10.3201/eid2411.180326

# Effects of Pneumococcal Conjugate Vaccine on Genotypic Penicillin Resistance and Serotype Changes, Japan, 2010–2017

## Technical Appendix

**Technical Appendix Table 1.** Serotypes, penicillin-resistant and macrolide-resistant genotypes for all isolates (n = 2,849) from patients with invasive pneumococcal disease, Japan, April 2010–March 2017

| | | Macrolide resistance† | | | |
|---|---|---|---|---|---|
| | Penicillin resistance | Non | *erm*(B) | *mef*(A) | *mef*(A) and *erm*(B) |
| Serotype (n) | genotype* | n = 258 | n = 1,704 | n = 557 | n = 330 |
| 4 (58) | gPISP (*pbp2x*) | | | 1 | |
| | gPSSP | 7 | 2 | 48 | |
| 6B (237) | gPRSP | | 117 | 23 | 10 |
| | gPISP (*pbp1a+2x*) | 2 | 14 | 26 | 2 |
| | gPISP (*pbp2x+2b*) | | 6 | 2 | |
| | gPISP (*pbp2x*) | | 26 | 7 | |
| | gPSSP | 2 | | | |
| 9V (31) | gPRSP | | 1 | | |
| | gPISP (*pbp2x*) | | 5 | 24 | |
| | gPSSP | | 1 | | |
| 14 (111) | gPRSP | 3 | 31 | 8 | |
| | gPISP (*pbp1a+2x*) | | 54 | 6 | 1 |
| | gPISP (*pbp2x+2b*) | | 4 | 1 | |
| | gPISP (*pbp2x*) | | 2 | | |
| | gPSSP | 1 | | | |
| 18C (15) | gPSSP | 9 | 2 | 4 | |
| 19F (107) | gPRSP | | 10 | 80 | 4 |
| | gPISP (*pbp1a+2x*) | | | 3 | 2 |
| | gPISP (*pbp1a+2b*) | | 1 | | |
| | gPISP (*pbp2x+2b*) | | | 1 | |
| | gPISP (*pbp2x*) | | 3 | 2 | |
| | gPSSP | 1 | | | |
| 23F (128) | gPRSP | 2 | 41 | 22 | 57 |
| | gPISP (*pbp1a+2x*) | | 3 | | |
| | gPISP (*pbp2x+2b*) | | 2 | | |
| | gPISP (*pbp2x*) | | | 1 | |
| 1 (36) | gPSSP | 11 | 9 | 16 | |
| 3 (314) | gPRSP | | 1 | | 1 |
| | gPISP (*pbp2x*) | 15 | 279 | 2 | |
| | gPISP (*pbp2b*) | | 1 | | |
| | gPSSP | 11 | 3 | 1 | |
| 5 (1) | gPSSP | | 1 | | |
| 6A (58) | gPRSP | 3 | 25 | 6 | 7 |
| | gPISP (*pbp1a+2x*) | 2 | 3 | 1 | |
| | gPISP (*pbp2x*) | 1 | 6 | 2 | |
| | gPSSP | 2 | | | |
| 7F (60) | gPISP (*pbp2x*) | | 4 | | |
| | gPSSP | 15 | 28 | 13 | |
| 19A (290) | gPRSP | | 1 | 1 | 89 |
| | gPISP (*pbp1a+2x*) | 1 | 7 | 1 | 28 |
| | gPISP (*pbp2x+2b*) | | | | 3 |
| | gPISP (*pbp2x*) | 4 | 3 | 51 | 85 |
| | gPSSP | 4 | 7 | 4 | 1 |
| 8 (2) | gPSSP | 2 | | | |
| 10A (123) | gPISP (*pbp1a+2x*) | 8 | 3 | | 1 |
| | gPISP (*pbp2x+2b*) | | 1 | | |
| | gPISP (*pbp2x*) | 9 | 86 | 4 | |
| | gPSSP | 2 | 9 | | |

| Serotype (n) | Penicillin resistance genotype* | Macrolide resistance† | | | |
|---|---|---|---|---|---|
| | | Non | *erm*(B) | *mef*(A) | *mef*(A) and *erm*(B) |
| | | n = 258 | n = 1,704 | n = 557 | n = 330 |
| 11A (67) | gPISP (*pbp1a+2x*) | | | 2 | |
| | gPISP (*pbp2x*) | 10 | | 3 | 26 |
| | gPSSP | 2 | 1 | 23 | |
| 12F (108) | gPISP (*pbp2x+2b*) | | 2 | | |
| | gPISP (*pbp2b*) | | 98 | | |
| | gPSSP | 4 | 4 | | |
| 15B (68) | gPRSP | | 1 | 1 | |
| | gPISP (*pbp1a+2x*) | | 12 | | |
| | gPISP (*pbp2x*) | | 50 | 1 | 1 |
| | gPSSP | 1 | | 1 | |
| 20 (23) | gPSSP | 3 | 19 | 1 | |
| 22F (169) | gPISP (*pbp1a+2x*) | | | 1 | |
| | gPISP (*pbp2x*) | 43 | 85 | 30 | |
| | gPSSP | 7 | 3 | | |
| 33F (42) | gPISP (*pbp2x*) | | | 5 | |
| | gPSSP | 2 | 34 | 1 | |
| 6C (154) | gPRSP | | 1 | 1 | |
| | gPISP (*pbp1a+2x*) | | 1 | | |
| | gPISP (*pbp2x+2b*) | 16 | 22 | 18 | 1 |
| | gPISP (*pbp2x*) | 4 | 62 | 25 | |
| | gPSSP | 2 | 1 | | |
| 6D (2) | gPRSP | | 1 | 1 | |
| 7C (10) | gPSSP | | 10 | | |
| 13 (1) | gPRSP | | 1 | | |
| 15A (144) | gPRSP | | 77 | | |
| | gPISP (*pbp1a+2x*) | | 56 | | |
| | gPISP (*pbp1a+2b*) | | 7 | | |
| | gPISP (*pbp2x*) | | 2 | | 1 |
| | gPSSP | | | 1 | |
| 15C (53) | gPRSP | | 2 | | |
| | gPISP (*pbp1a+2x*) | | 9 | 1 | |
| | gPISP (*pbp2x*) | | 40 | 1 | |
| 16F (8) | gPRSP | | | 4 | |
| | gPISP (*pbp1a+2x*) | | | 2 | |
| | gPSSP | 2 | | | |
| 18B (1) | gPSSP | 1 | | | |
| 21 (2) | gPISP(*pbp2x*) | 1 | | 1 | |
| 23A (112) | gPRSP | | 1 | | |
| | gPISP (*pbp2x+2b*) | 1 | 104 | | |
| | gPISP (*pbp2x*) | | 1 | | 3 |
| | gPSSP | 2 | | | |
| 23B (8) | gPRSP | 1 | | | |
| | gPISP (*pbp2x+2b*) | 2 | | | |
| | gPISP (*pbp2x*) | 1 | | | |
| | gPSSP | 2 | 1 | 1 | |
| 24F (100) | gPISP (*pbp1a+2x*) | | 1 | | |
| | gPISP (*pbp2x*) | | 2 | | |
| | gPSSP | | 97 | | |
| 24B (17) | gPSSP | | 17 | | |
| 28A (1) | gPSSP | 1 | | | |
| 31 (5) | gPSSP | 5 | | | |
| 34 (32) | gPRSP | | 1 | | |
| | gPISP(*pbp2x*) | 3 | 1 | 9 | |
| | gPSSP | 5 | 13 | | |
| 35B (106) | gPRSP | 6 | 9 | 33 | 7 |
| | gPISP (*pbp1a+2x*) | | 1 | | |
| | gPISP (*pbp2x*) | | 42 | 1 | |
| | gPSSP | | 7 | | |
| 37 (8) | gPSSP | 5 | 2 | 1 | |
| 38 (37) | gPISP (*pbp2x+2b*) | | 1 | | |
| | gPISP (*pbp2x*) | 1 | | 22 | |
| | gPSSP | 8 | | 5 | |

**1a*, *2x*, and *2b* in parenthesis indicate abnormal *pbp1a*, *pbp2x*, and *pbp2b* genes, respectively.
†Each gene mediates macrolide resistance such as clarithromycin (CLR) and azithromycin (AZM).

**Technical Appendix Table 2.** $MIC_{50}$, $MIC_{90}$, and MIC range of 6 intravenous antimicrobial agents for isolates from patients with invasive pneumococcal disease from April 2014 to March 2017 (n = 1229)

| | Concentration (µg/mL) | | |
|---|---|---|---|
| Antimicrobial agent | $MIC_{50}$ | $MIC_{90}$ | MIC range |
| Penicillin | 0.063 | 2 | 0.016–4 |
| Ampicillin | 0.063 | 2 | 0.016–8 |
| Cefotaxime | 0.25 | 1 | 0.008–8 |
| Meropenem | 0.008 | 0.5 | 0.008–1 |
| Levofloxacin | 2 | 2 | 0.5–32 |
| Vancomycin | 0.5 | 1 | 0.5–1 |

**Technical Appendix 3**. Serotypes, resistance genotypes, and multilocus sequence types for all isolates (n = 2,849) from patients with invasive pneumococcal disease, Japan, April 2010 - March 2017

| Serotype | Resistance genotype† | Clonal complex (n) | Sequence type (n) |
|---|---|---|---|
| PCV13 | | | |
| 4 | gPISP(*2x*) | 81 (1) | 899 (1) |
| | gPSSP | 81 (51); 3784 (1); 5902 (1); none (4) | 246 (43) 695 (1) 899 (5) 7880 (1) 8649 (1); 7776 (1); 13576 (1)*; 5872 (4) |
| 6B | gPRSP | 156 (84); 81 (28); none (11); 9789 (9); 2224 (8); 2924 (5); Singleton (3); 242 (2) | 90 (66) 95 (3) 273 (5) 5497 (3) 7417 (1) 7827 (1) 7831 (2) 7870 (1) 13591 (1)* 13593 (1)*; 81 (1) 282 (1) 902 (19) 2923 (1) 3849 (1) 7492 (2) 8345 (1) 8644 (1) 8648(1); 5232 (10) 7967 (1); 2756 (3) 3787 (6); 2224 (7) 7835 (1); 6413 (5); 5244 (3); 242 (2) |
| | gPISP(*1a*+*2x*) | 156 (23); 2224 (14); 7834 (4); 2924 (2); Singleton (1) | 2983 (16) 5497 (5) 7824 (2); 2224 (13) 7826 (1); 7834 (4); 6183 (2); 13046 (1) |
| | gPISP(*2x*+*2b*) | 81 (5); 156 (1); 2924 (1); 7834 (1) | 902 (3) 7965(1) 12044 (1); 5830 (1); 8348 (1); 7834 (1) |
| | gPISP(*2x*) | 81 (15); 156 (12); 2924 (4); 7834 (1); Singleton (1) | 902 (1) 2923 (11) 5245 (1) 6430 (2); 273 (1) 2983 (7) 7839 (2) 10194 (2); 2924 (4); 7834 (1); 13048 (1) |
| | gPSSP | 2224 (1); 7585 (1) | 2224(1); 6771(1) |
| 9V | gPRSP | 156 (1) | 166 (1) |
| | gPISP(*2x*) | 156 (29) | 280 (18) 5231 (11) |
| | gPSSP | 156 (1) | 280 (1) |
| 14 | gPRSP | 343 (30); 156 (5); 15 (2); 230 (1); 63 (1); 199 (1); 320 (1); Singleton (1) | 343 (23) 554 (2) 3388 (1) 7837 (1) 7971 (2) 7974 (1); 156 (2) 5493 (3); 2922 (2); 230 (1); 782 (1); 876(1); 236 (1); 8642 (1) |
| | gPISP(*1a*+*2x*) | 15 (41); 230 (19); 3111 (1) | 13 (3) 2922 (38); 5240 (17) 7966 (1) 7973 (1); 13571 (1)* |
| | gPISP(*2x*+*2b*) | 343 (4); 15 (1) | 343 (2); 554(2); 15 (1) |
| | gPISP(*2x*) | 156 (2) | 124 (1) 7972 (1) |
| | gPSSP | 156 (1) | 124 (1) |
| 18C | gPSSP | 3594 (14); 870 (1) | 3594 (13) 7829 (1); 870 (1) |
| 19F | gPRSP | 320 (88); 156 (3); 63 (1); 81 (1); 242 (1) | 236 (77) 926 (1) 1421 (1) 1428 (1) 1464(1) 4472 (1) 7873 (2) 7991 (1) 8341 (1) 8344 (1) 13044 (1); 7993 (1) 8352 (2); 10211 (1); 81 (1); 8650 (1) |
| | gPISP(1a+2x) | 156 (2); 320 (1); 3111 (1); Singleton (1) | 1263 (1) 7993 (1); 926 (1); 3111 (1); 12001(1) |
| | gPISP(*1a*+*2b*) | 156 (1) | 7993 (1) |
| | gPISP(*2x*+*2b*) | 320 (1) | 236 (1) |
| | gPISP(*2x*) | 320 (3); 177 (1); 2924 (1) | 236 (2) 257 (1); 179 (1); 6183 (1) |
| | gPSSP | 251 (1) | 654 (1) |
| 23F | gPRSP | 242 (59); 2924 (55); 156 (6); 81 (1); 5832 (1) | 242 (48) 1435 (3) 1444 (1) 3589 (1) 7386 (1) 7832 (1) 7968 (1) 8343 (1) 10409 (1) 13575 (1)*; 1437 (51) 6434 (1) 7836 (1) 7872 (2); 338 (5) 2777 (1); 1623 (1); 7989 (1) |
| | gPISP(*1a*+*2x*) | 63 (2); 242 (1) | 63 (1) 8643 (1); 5844 (1) |
| | gPISP(*2x*+*2b*) | 156 (2) | 338 (1) 5242 (1) |
| | gPISP(*2x*) | 242 (1) | 242 (1) |
| 1 | gPSSP | 306 (34); 217 (2) | 306 (33) 5239 (1); 5002 (2) |
| 3 | gPRSP | 156 (1); 242 (1) | 166 (1); 242 (1) |
| | gPISP(*2x*) | 180 (292); 99 (1); 113 (1); 156 (1); 242 (1) | 180 (277) 5234 (5) 7875 (1) 7877 (1) 7878 (1) 8646 (1) 8652 (1) 10025 (1) 10420 (2) 11192 (1) 11997 (1); 99 (1); 13045 (1); 1263 (1); 7386 (1) |
| | gPISP(*2b*) | 1527 (1) | 13574 (1)* |
| | gPSSP | 180 (13); Singleton (2) | 180 (10) 505 (1) 2570 (1) 12002 (1); 10216 (1) 12005 (1) |
| 5 | gPSSP | 289 (1) | 289 (1) |
| 6A | gPRSP | 9789 (25); 81 (12); 156 (1); 3115 (1); Singleton (2) | 2756 (17) 3113 (1) 6432 (2) 6437 (2) 7825 (2) 8350 (1); 81 (6) 282 (5) 13572 (1)*; 90 (1); 3115 (1); 5244 (1) 7871 (1) |
| | gPISP(1a+2x) | 9789 (3); 156 (1); 2924 (1); Singleton (1) | 3113 (2) 3787 (1); 5833 (1); 6183 (1); 11998 (1) |

| Serotype | Resistance genotype† | Clonal complex (n) | Sequence type (n) |
|---|---|---|---|
| | gPISP(2x) | 9789 (7); 81 (2) | 3113 (5) 3787 (1) 7969 (1); 2923 (2) |
| | gPSSP | 9789 (1); Singleton (1) | 4271 (1); 8347 (1) |
| 7F | gPISP(2x) | 191 (4) | 191 (4) |
| | gPSSP | 191 (54); 218 (2) | 191 (54); 405 (2) |
| 19A | gPRSP | 3111 (86); 156 (1); 320 (4) | 3111 (85) 9045 (1); 156 (1); 320 (4) |
| | gPISP(*1a*+*2x*) | 3111 (28); 2331 (9) | 3111 (27) 7988 (1); 2331 (1) 5237 (8) |
| | gPISP(*2x*+*2b*) | 3111 (3) | 3111 (3) |
| | gPISP(*2x*) | 3111 (89); 2331 (54) | 3111 (88) 7988 (1); 2331 (52) 13047 (2) |
| | gPSSP | 2331 (13); 3111 (3) | 2331 (9) 5842 (4); 3111 (2) 8339 (1) |
| NVT | | | |
| 8 | gPSSP | 156 (1); none (1) | 11999 (1); 6022 (1) |
| 10A | gPISP(*1a*+*2x*) | 156 (9); 113 (2); Singleton (1) | 1263 (8) 7773 (1); 5236 (2); 3078 (1) |
| | gPISP(*2x*+*2b*) | 156 (1) | 5246 (1) |
| | gPISP(*2x*) | 113 (84); 156 (15) | 5236 (84); 1263 (11) 3395 (1) 7773 (1) 7830 (2) |
| | gPSSP | 113 (9); 156 (2) | 5236 (9); 1263 (1) 12051 (1) |
| 11A | gPISP(*1a*+*2x*) | 99 (2) | 99 (2) |
| | gPISP(*2x*) | 99 (38); 62 (1) | 99 (30) 7153 (7) 7811 (1); 1760 (1) |
| | gPSSP | 99 (23); 62 (3) | 99 (21) 7153 (1) 8651 (1); 62 (2) 1012 (1) |
| 12F | gPISP(*2x*+*2b*) | 1527 (2) | 4846 (2) |
| | gPISP(*2b*) | 1527 (98) | 4846 (98) |
| | gPSSP | 1527 (7); 989 (1) | 6945 (6) 12003 (1); 989 (1) |
| 15B | gPRSP | 81 (1); 242 (1) | 83 (1); 242 (1) |
| | gPISP(*1a*+*2x*) | 199 (12) | 199 (12) |
| | gPISP(*2x*) | 199 (52) | 199 (51) 5609 (1) |
| | gPSSP | 199 (2) | 199 (2) |
| 20 | gPSSP | 4745 (20); 99 (1); 235 (1); 5349 (1) | 4745 (16) 7828 (4); 99 (1); 235 (1); 5190 (1) |
| 22F | gPISP(*1a*+*2x*) | 433 (1) | 4553 (1) |
| | gPISP(*2x*) | 433 (147); 113 (11) | 433 (145) 819 (1) 10023 (1); 5236 (2) 7158 (9) |
| | gPSSP | 433 (5); 113 (2); 2572 (1); 3594 (1); none (1) | 433 (5); 7158 (1) 8353 (1); 5496 (1); 3594 (1); 3465 (1) |
| 33F | gPISP(*2x*) | 100 (5) | 100 (5) |
| | gPSSP | 717 (34); 62 (2); 100 (1) | 717 (34); 673 (2); 100 (1) |
| 6C | gPRSP | 156 (1); 5832 (1) | 8352 (1); 5832 (1) |
| | gPISP(*1a*+*2x*) | 2924 (1) | 12080 (1) |
| | gPISP(*2x*+*2b*) | 156 (30); 5832 (24); 315 (2); Singleton (1) | 5241 (29) 12081 (1); 5025 (2) 5832 (20) 7384 (2); 386 (2); 9025 (1) |
| | gPISP(*2x*) | 2924 (59); 81 (23); 7781 (4); 156 (2); 9789 (2); 242 (1) | 2924 (37) 6183 (16) 7838 (1)7879 (1) 9336 (2)12080 (1)12082 (1); 2923 (23); 7788 (2) 7876 (2); 4233 (1) 13587 (1)*; 3787 (2); 242 (1) |
| | gPSSP | 156 (1); 473 (1); 7781 (1) | 8645 (1); 473 (1); 2789 (1) |
| 6D | gPRSP | 81 (1); 156 (1) | 282 (1); 90 (1) |
| 7C | gPSSP | 2758 (10) | 2758 (10) |
| 13 | gPRSP | 156 (1) | 10303 (1) |
| 15A | gPRSP | 63 (77) | 63 (73) 2105 (1) 2771 (1) 8354 (1) 12000 (1) |
| | gPISP(*1a*+*2x*) | 63 (56) | 63 (42) 7874 (13) 10211 (1) |
| | gPISP(*1a*+*2b*) | 63 (6); Singleton (1) | 63 (6); 8346 (1) |
| | gPISP(*2x*) | 292 (2) 113 (1) | 292 (2); 7992 (1) |
| | gPSSP | 292 (1) | 292 (1) |
| 15C | gPRSP | 81 (2) | 83 (1) 6138 (1) |
| | gPISP(*1a*+*2x*) | 199 (10) | 199 (9) 1674 (1) |
| | gPISP(*2x*) | 199 (41) | 199 (39) 9222 (1) 12073 (1) |
| 16F | gPRSP | 3117 (4) | 8351 (4) |
| | gPISP(*1a*+*2x*) | 3117 (2) | 3117 (2) |
| | gPSSP | 3117 (2) | 3117 (2) |
| 18B | gPSSP | 3594 (1) | 3594 (1) |
| 21 | gPISP(*2x*) | 1381 (2) | 1233 (2) |
| 23A | gPRSP | 156 (1) | 9619 (1) |
| | gPISP(*2x*+*2b*) | 156 (105) | 338 (57) 2612 (1) 3437 (1) 5242 (39) 5246 (5) 6685 (1) 8340 (1) |
| | gPISP(*2x*) | 156 (4) | 338 (1) 10000 (3) |
| | gPSSP | 439 (2) | 42 (1) 438 (1) |
| 23B | gPRSP | 156 (1) | 2372 (1) |
| | gPISP(*2x*+*2b*) | 156 (2) | 1373 (2) |
| | gPISP(*2x*) | 439 (1) | 439 (1) |
| | gPSSP | 439 (3); 63 (1) | 439 (3); 63 (1) |
| 24 | gPISP(*1a*+*2x*) | 230 (1) | 230 (1) |
| | gPISP(*2x*) | 2572 (2) | 2572 (2) |
| | gPSSP | 2572 (112); 4982 (2) | 2572 (62 )5496 (50); 4982 (2) |

| Serotype | Resistance genotype† | Clonal complex (n) | Sequence type (n) |
|---|---|---|---|
| 28A | gPSSP | 546 (1) | 11601 (1) |
| 31 | gPSSP | 7800 (5) | 11184 (5) |
| 34 | gPRSP | 15 (1) | 9395 (1) |
| | gPISP(*2x*) | none (12); 15 (1) | 3116 (12); 7388 (1) |
| | gPSSP | 15 (13); none (5) | 1439 (1) 7388 (12); 3116 (5) |
| 35B | gPRSP | 558 (53); 156 (1); Singleton (1) | 558 (49) 1204 (2) 7809 (2); 156 (1); 7990 (1) |
| | gPISP(*1a*+*2x*) | 1816 (1) | 2755 (1) |
| | gPISP(*2x*) | 1816 (43) | 2755 (42) 12004 (1) |
| | gPSSP | 1816 (7) | 2755 (7) |
| 37 | gPSSP | 447 (8) | 447 (6) 7970 (2) |
| 38 | gPISP(*2x*+*2b*) | none (1) | 7833 (1) |
| | gPISP(*2x*) | 6429 (23) | 6429 (23) |
| | gPSSP | 393 (12); 6429 (1) | 393 (12); 6429 (1) |

*STs newly identified in this study.
†*1a, 2x*, and *2b* in the parenthesis indicate *pbp1a*, *pbp2x* and *pbp2b genes*, respectively. PBPs (PBP1A, PBP2X, and PBP2B) involved in peptidoglycan synthesis are encoded by these genes. Amino acid substitutions within or near each PBP's conserved amino acid motifs were identified in resistance strains with various combinations of abnormal genes.

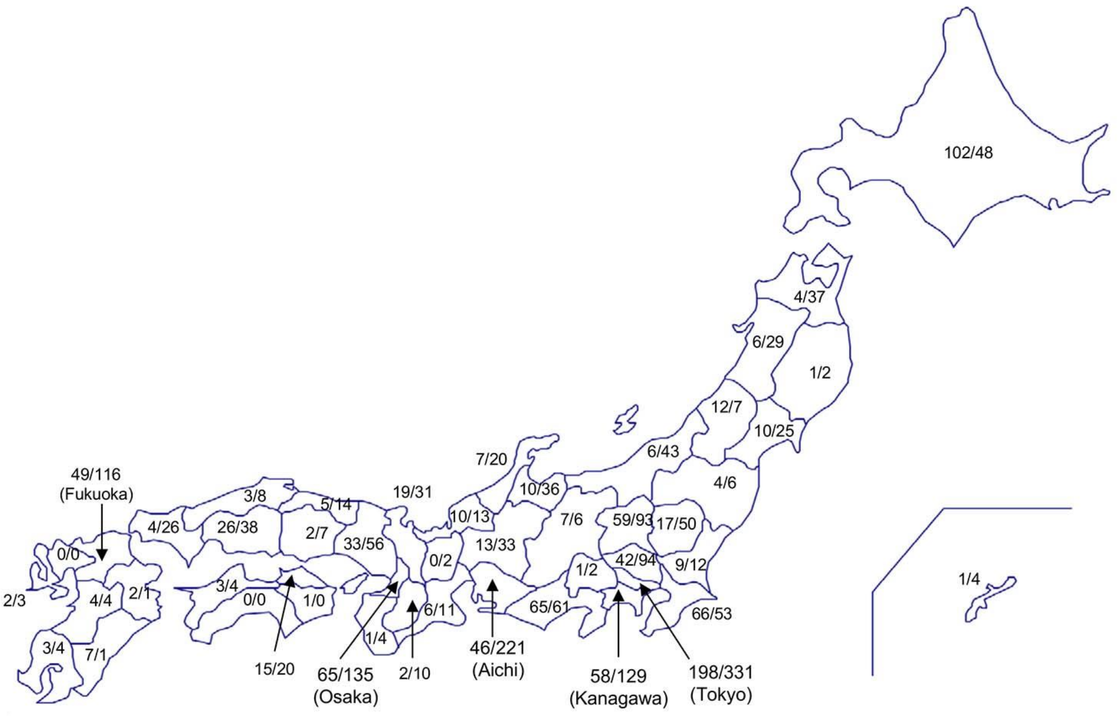


**Technical Appendix Figure 1.** Distribution of isolates collected by the surveillance for invasive pneumococcal disease in Japan from April 2010 to March 2017. Numbers of isolates obtained from children/adults are shown for each locality on the map.

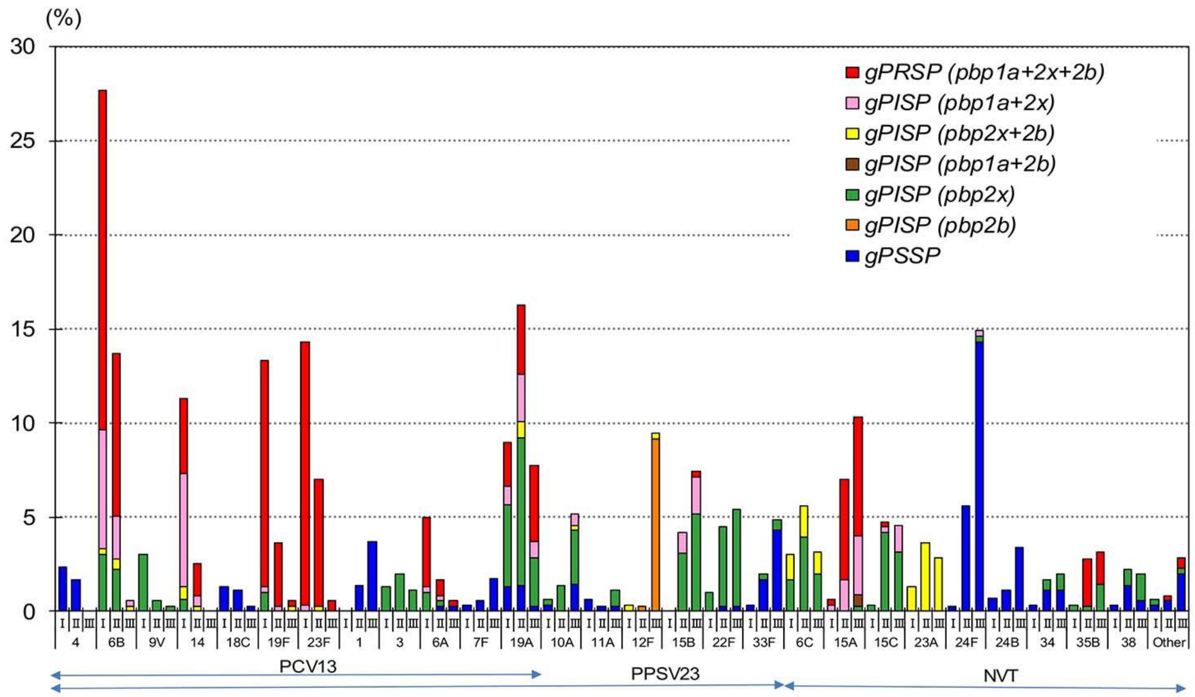


**Technical Appendix Figure 2.** Yearly changes of serotypes and penicillin-resistant genotypes among isolates from children with invasive pneumococcal disease in Japan from April 2010 to March 2017. I, II, and III on horizontal axis represent three surveillance periods as follows: pre-PCV7 period, from April 2010 through March 2011; PCV7 period, from April 2011 to March 2014; and PCV13 period, from April 2014 through March 2017. gPSSP, genotypic penicillin-susceptible *Streptococcus pneumoniae*; gPISP, genotypic penicillin-intermediate resistant *S. pneumoniae*; gPRSP, genotypic penicillin-resistant *S. pneumoniae.* Parentheses enclose mutated *pbp* genes, *pbp1a, pbp2x,* and/or *pbp2b,* that mediate penicillin resistance.

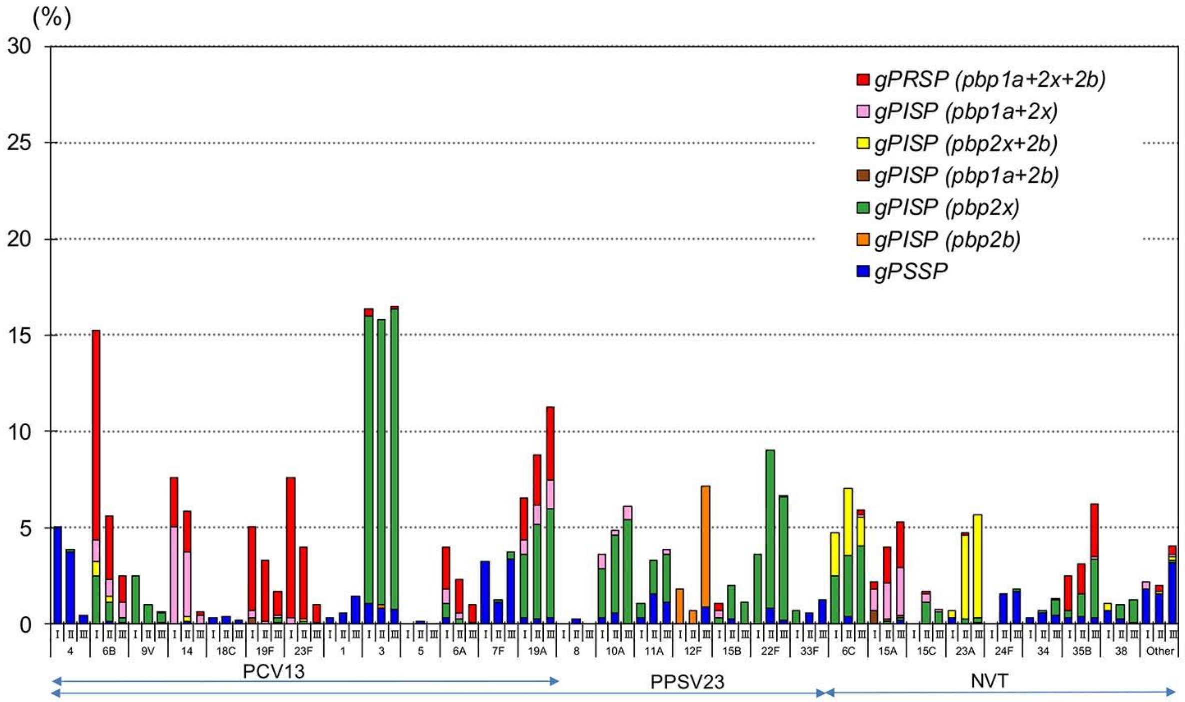


**Technical Appendix Figure 3.** Yearly changes of serotypes and penicillin-resistant genotypes among isolates from adults with invasive pneumococcal disease in Japan from April 2010 to March 2017. I, II, and III on horizontal axis represent three surveillance periods as follows: pre-PCV7 period, before vaccination to children, from April 2010 to March 2011; PCV7 period, after PCV7 vaccination to children, from April 2011 to March 2014; and PCV13 period, with PCV7 replaced by PCV13 vaccination in children, from April 2014 to March 2017. gPSSP, genotypic penicillin-susceptible *Streptococcus pneumoniae*; gPISP, genotypic penicillin-intermediate resistant *S. pneumoniae*; gPRSP, genotypic penicillin-resistant *S. pneumoniae.* Parentheses enclose mutated *pbp* genes, *pbp1a, pbp2x,* and/or *pbp2b,* that mediate penicillin resistance.

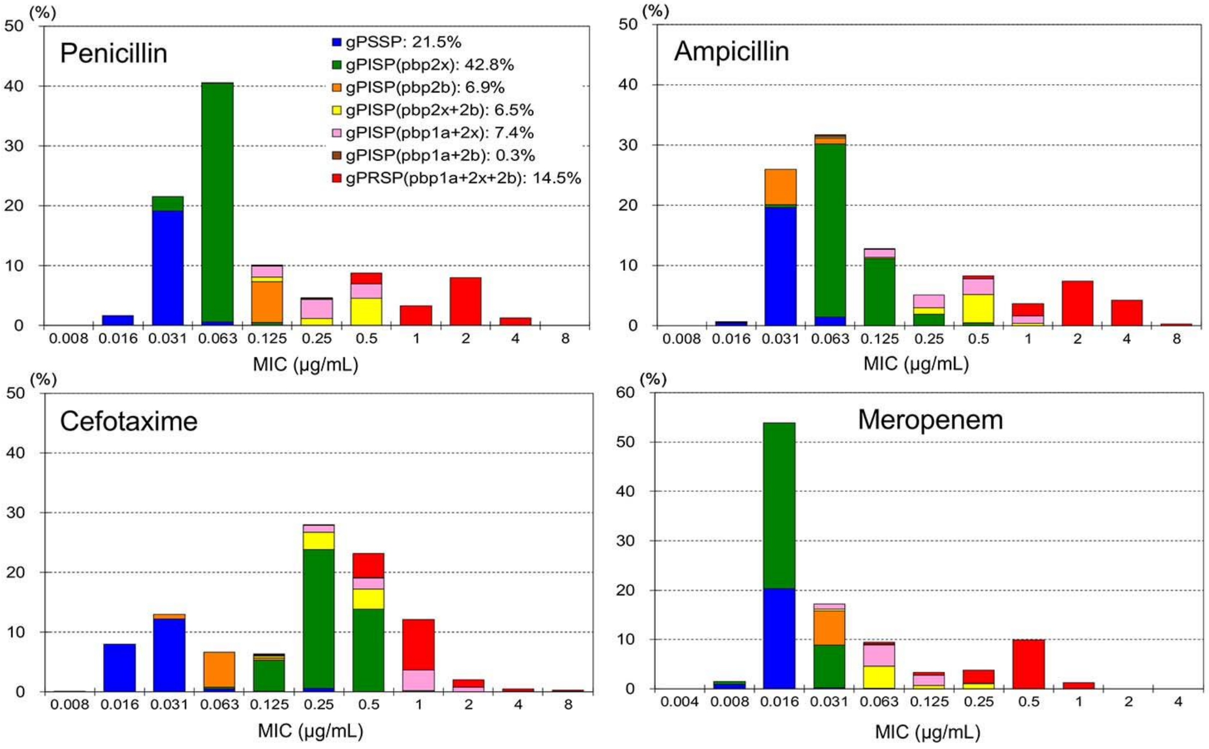


**Technical Appendix Figure 4.** Distribution of susceptibilities to 4 parenteral β-lactam antibiotics, penicillin, ampicillin, cefotaxime, and meropenem, by genotypes of isolates from patients with invasive pneumococcal disease in Japan from April 2014 to March 2017 (n = 1229). gPSSP indicates genotypic penicillin-susceptible *Streptococcus pneumoniae*; gPISP indicates genotypic penicillin-intermediate-resistant *S. pneumoniae*; and gPRSP indicates genotypic penicillin-resistant *S. pneumoniae*. Parentheses enclose mutated *pbp* genes *pbp1a, pbp2x,* and/or *pbp2b,* which mediate penicillin resistance.

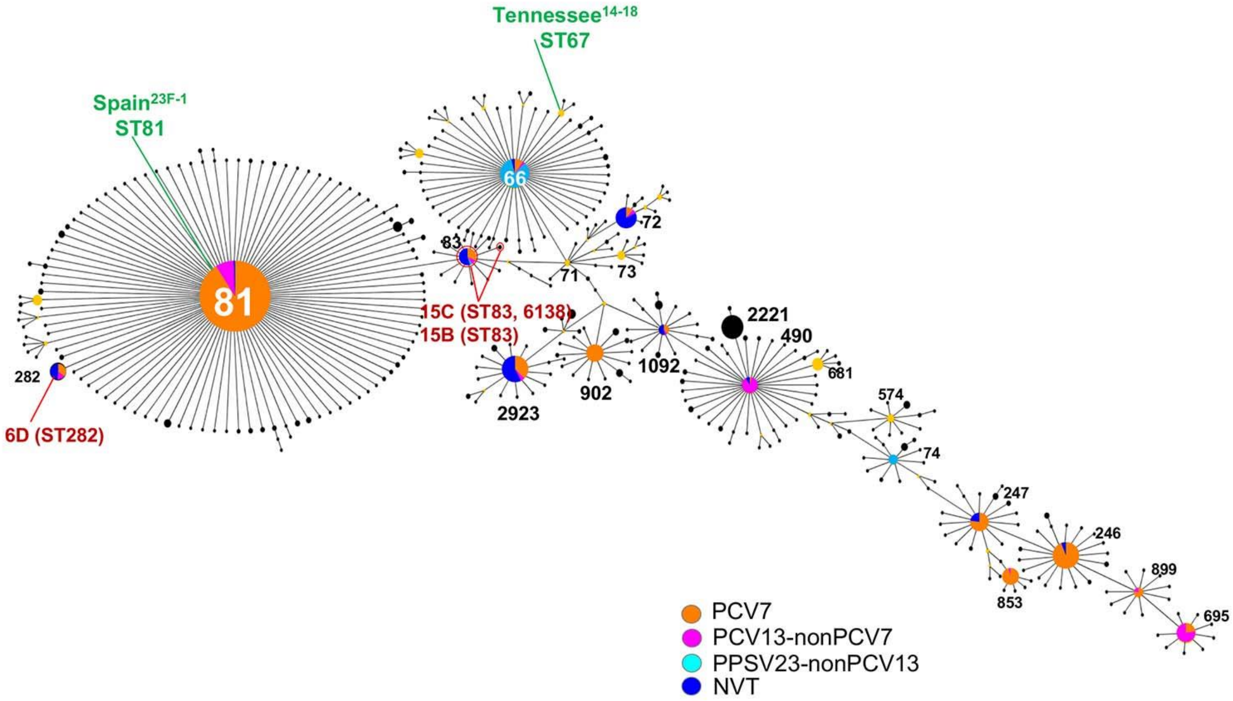


**Technical Appendix Figure 5.** Details of clonal complex (CC) 81 (n = 1858) included 420 sequence types (STs) from the Multilocus Sequence Typing Web site (https://pubmlst.org/spneumoniae/) (download October/02/2017). Data include our own from the present study (n = 143). STs of 3 gPRSPs identified in serotypes 6D, 15B, and 15C (in red) were derived from ST81, belonging to CC81. The Pneumococcal Molecular Epidemiology Network (PMEN) clones identified in CC81 also are shown (in green).
